# Deep learning-based dual-energy subtraction synthesis from single-energy kV x-ray fluoroscopy for markerless tumor tracking

**DOI:** 10.1007/s11517-025-03432-9

**Published:** 2025-08-27

**Authors:** Jiaoyang Wang, Kei Ichiji, Yuwen Zeng, Xiaoyong Zhang, Yoshihiro Takai, Noriyasu Homma

**Affiliations:** 1 Graduate School of Biomedical Engineering, Tohoku University, Aoba-6-3 Aramaki, 980-8579 Sendai, Miyagi Japan; 2 Tohoku University Graduate School of Medicine, Tohoku University, 1-1 Seiryo-machi, 980-8575 Sendai, Miyagi Japan; 3 National Institute of Technology, Sendai College, 4-16-1, 989-3128 Sendai, Miyagi Japan; 4 Southern Tohoku BNCT Research Center, Yatsuyamada, 7 Chome 10, 963-8052 Koriyama, Fukushima Japan

**Keywords:** Radiation therapy, Markerless tumor tracking, Dual-energy subtraction, Deep learning

## Abstract

**Abstract:**

Markerless tumor tracking in x-ray fluoroscopic images is an important technique for achieving precise dose delivery for moving lung tumors during radiation therapy. However, accurate tumor tracking is challenging due to the poor visibility of the target tumor overlapped by other organs such as rib bones. Dual-energy (DE) x-ray fluoroscopy can enhance tracking accuracy with improved tumor visibility by suppressing bones. However, DE x-ray imaging requires special hardware, limiting its clinical use. This study presents a deep learning-based DE subtraction (DES) synthesis method to avoid hardware limitations and enhance tracking accuracy. The proposed method employs a residual U-Net model trained on a simulated DES dataset from a digital phantom to synthesize DES from single-energy (SE) fluoroscopy. Experimental results using a digital phantom showed quantitative evaluation results of synthesis quality. Also, experimental results using clinical SE fluoroscopic images of ten lung cancer patients showed improved tumor tracking accuracy using synthesized DES images, reducing errors from 1.80 to 1.68 mm on average. The tracking success rate within a 25% movement range increased from 50.2% (SE) to 54.9% (DES). These findings indicate the feasibility of deep learning-based DES synthesis for markerless tumor tracking, offering a potential alternative to hardware-dependent DE imaging.

**Graphical abstract:**

The U-Net model was first trained on simulated single-energy and dual-energy subtraction image pairs, then applied to synthesize DES from clinical X-ray images for improving tumor tracking
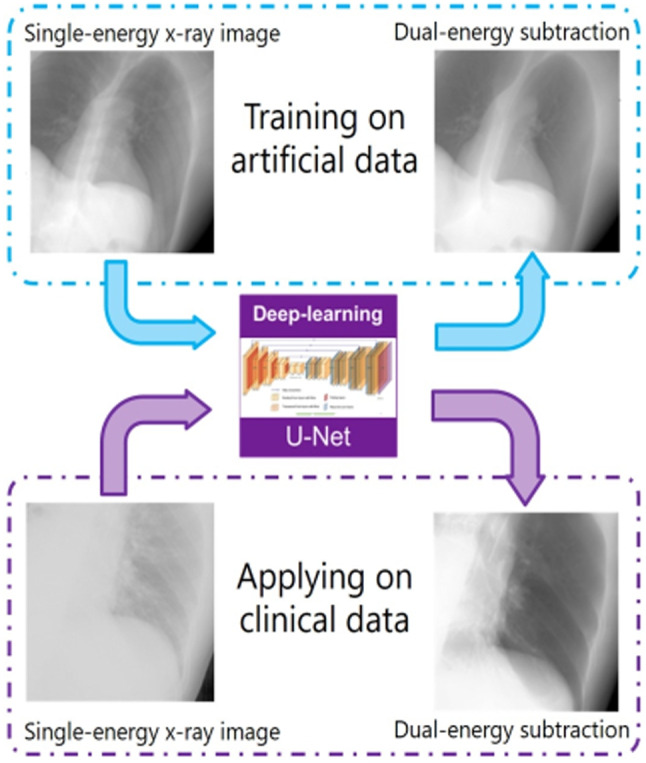

## Introduction

Lung cancer is the leading cause of cancer-related deaths worldwide [1], and there is a great demand to develop effective treatment methods for lung cancer. Radiation therapy is one potential treatment approach by delivering high-energy radiation beams at tumor cells to kill or inhibit their proliferation. To ensure the accurate and precise delivery of radiation doses to the lung tumor moving with respiration, various real-time tumor tracking methods for beam adaptation have been reported [2, 3].

Kilo-volatage (kV) x-ray fluoroscopy is a popular imaging modality for monitoring moving tumors inside the patient’s body in real time and is clinically available by on-board imaging devices equipped on most radiotherapy machines. Conventionally, fiducial markers are implanted around internal tumors as a reference to accurately localize the tumor in x-ray images. However, marker implantation carries the risks of complications such as pneumothorax and marker migration [4, 5]. Therefore, researchers have been focusing on the development of markerless tumor tracking (MTT) methods, which aim to track tumors directly without fiducial markers. These methods include template matching-based approaches [6, 7], key-point-based tracking methods [8, 9], and machine learning-based methods [10, 11]. Although those MTT methods have demonstrated the potential to accurately track the tumors without markers, the MTT methods often face challenges in reduced tracking accuracy, particularly when overlapping internal organs such as bones obscure internal tumors in x-ray images, leading to poor visibility of the tumors [12, 13].

**Fig. 1:**
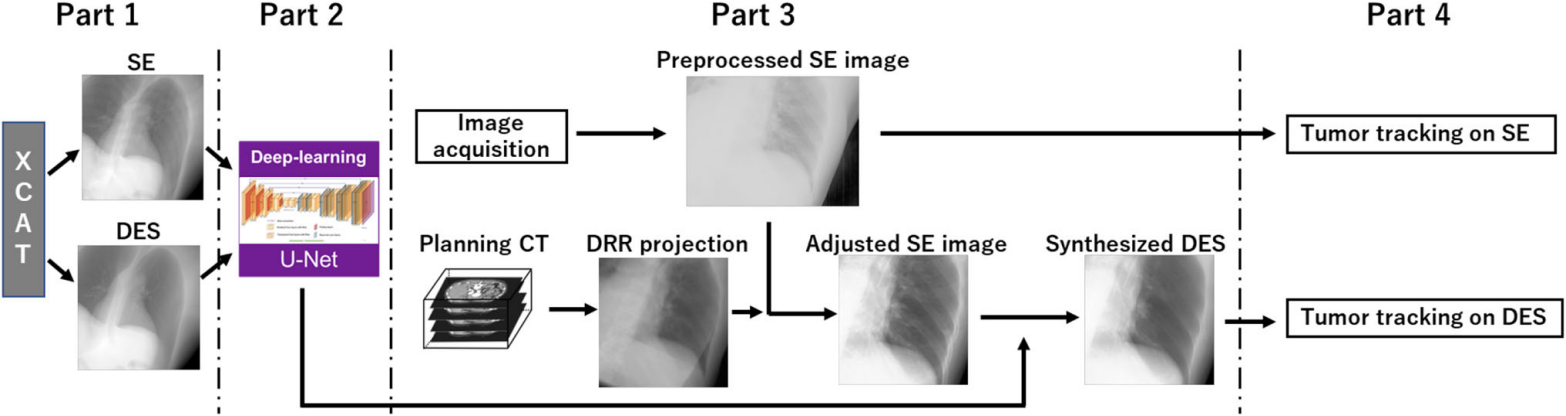
The workflow in this study. Part (1): training data preparation. Part (2): DL model training. Part (3): DES synthesis. Part (4): tumor tracking on SE and synthetic DES images

For mitigating the performance degradation of MTT, several studies have shown the capabilities of dual-energy (DE) x-ray fluoroscopy [14–18]. Generally, DE imaging consists of obtaining two x-ray images by two different energy levels (e.g., 120 kVp and 60 kVp). By taking a weighted logarithmic subtraction of the two x-ray images, dual-energy subtraction (DES) can suppress bone components and enhance soft tissue, thereby mitigating the effects of bone overlap. For instance, Patel et al. [14] demonstrated that DES images can improve tumor detectability and MTT accuracy with standard template matching compared to MTT on conventional single-energy (SE) x-ray fluoroscopic images. Unlike conventional SE x-ray fluoroscopy, DE x-ray fluoroscopy requires specific hardware to obtain paired images simultaneously at different energy levels during treatment. For example, Haytmyradov et al. [16] implemented a fast-switching DE fluoroscopic device, and Shi et al. [17] developed a prototype dual-layer flat panel detector which can provide DE images with a single-energy x-ray exposure. While these studies have demonstrated the potential of DE fluoroscopy for accurate tumor tracking, incorporating DE imaging into clinical practice remains challenging due to the installation costs of special hardware.

In recent years, deep learning (DL)-based synthesizing of medical images has achieved considerable success [19, 20]. DL-based synthesizing of DE images from SE images has also been investigated as a possible way to address the limitations of actual hardware-based DE imaging for non-tumor tracking applications [21–23]. For example, Lee et al. [21] proposed a U-net model-based method to predict DE chest x-ray radiography (DE-CXR) from SE-CXR images. Zhao et al. [22] applied a U-net model to synthesize DE computed tomography (DE-CT) from SE-CT data. Despite these studies suggest the feasibility of DL-based synthesis of DE fluoroscopy, the DL-based synthesis of DE fluoroscopy for tumor tracking in radiation therapy has not yet been investigated. A reason for this gap is that DE imaging applications for MTT in radiation therapy are still in the early developmental stage, leading to limited clinical availability and a shortage of DE imaging data for DL model training. Training a DL model to comprehend the relationship between SE and DE presents a significant challenge, necessitating a diverse training dataset that covers various clinical scenarios during radiation therapy treatment.

This study aims to investigate the feasibility of using a DL-based approach to synthesize DE fluoroscopy for MTT. We apply a residual U-net model to synthesize DES images from SE images for excluding bone structures. To address the challenges arising from the shortage of clinical DE fluoroscopic image data, we employ a digital phantom to generate DE fluoroscopic image data encompassing various scenarios for training our DL model. A workflow for the DES synthesis during treatment is also considered for presenting the capability of the DES synthesis integrated into clinical situations. Subsequently, we test the performance of MTT with the DES synthesis by using ten clinical cases obtained through stereotactic body radiation therapy. This analysis allows us to determine the potential benefits and contribution of applying DE fluoroscopy synthesis in a clinical scenario.

**Fig. 2:**
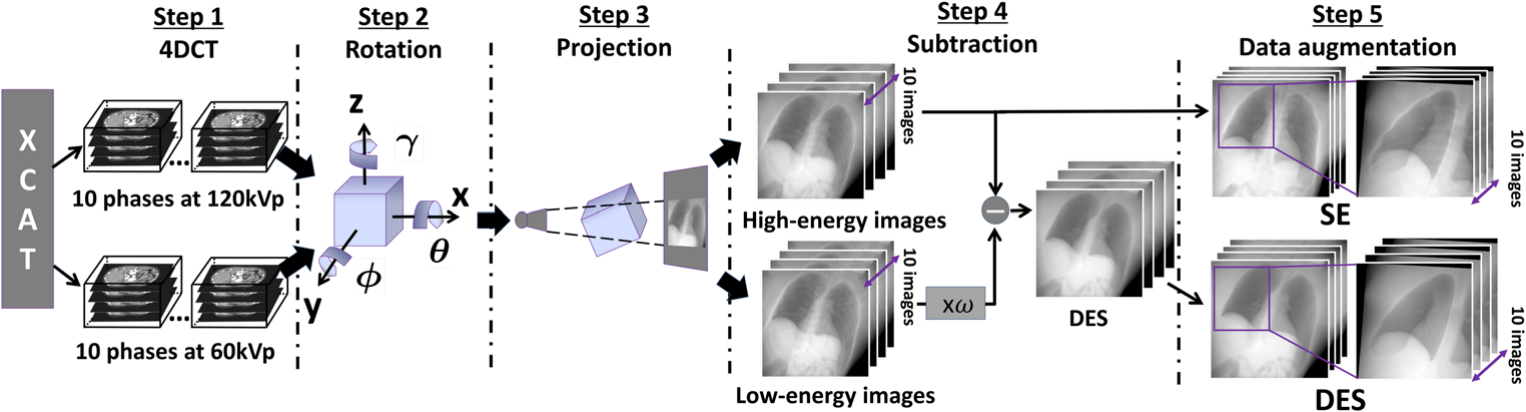
Schematic of training data preparation in Part 1. Five steps from generating DE-CT data to preparing SE and DES image pairs

## Materials and methods

As shown in Fig. 1, this study consists of four parts: (1) training data preparation: SE images and its corresponding reference DES images are generated from a digital phantom; (2) DL model training: a residual U-net model is trained using pairs of SE and DES as the input and output; (3) DES synthesis: clinical x-ray fluoroscopic images are adjusted and input to the trained model; Furthermore, (4) tumor tracking: template matching-based MTT is implemented to track the moving tumors on SE and the synthetic DES, respectively.

### Training dataset preparation

Part 1 is to generate SE and DES image pairs for the deep learning model to learn the relationship between SE and DES. Due to the lack of clinical DE data, we utilize digitally reconstructed radiographs (DRRs) generated from a digital phantom of four-dimensional CT data to simulate x-ray images taken at different energy levels.

The process described in this part includes generating CT data at varying energy levels, followed by simulating the path of x-rays through the patient’s body using a reconstruction method to produce the corresponding DRRs. By subtracting these DRRs acquired at different energy levels, we can obtain the DES images. The training data preparation process is illustrated in Fig. 2.

#### Step 1: CT data generation

To obtain the realistic x-ray fluoroscopic images at two different energy levels during a treatment, we first generate artificial four-dimensional CT (4DCT) data of thoracic and abdominal organs and tissues with respiratory motion by using the four-dimensional extended cardia-torso (XCAT) phantom [24]. Specifically, we produce two sets of 4D CT data from a single simulated anatomy: one at high-energy (HE) and the other at low-energy (LE) levels (120 kVp and 60 kVp, respectively), each consisting of 10 respiratory phases ($p=1,2,\dots ,10$), with three-dimensional coordinates $\varvec{r}=(x,y,z)$. We represent the CT data (at arbitrary energy level) with the mathematical notations $\varvec{V}_{\text {CT},p}(\varvec{r})$, which including the data at high-energy $\varvec{V}_{\text {HE-CT},p}(\varvec{r})$ and low-energy levels $\varvec{V}_{\text {LE-CT},p}(\varvec{r})$.

#### Step 2: CT data rotation

The generated CT data is rotated to cover a wide range of treatment scenarios encountered in clinical practice. That is, rotation along with the three-dimensional coordinate $\varvec{r}$ is performed to imitate various imaging conditions during treatment sessions, including gantry and couch angles. Using elemental rotation matrices, the generated volumetric CT data are rotated three-dimensionally as:

1 $$\begin{aligned} \varvec{V}_{\text {CT}, p, \theta , \phi , \gamma }(\varvec{r})=\varvec{V}_{\text {CT},p}(\varvec{\varvec{R}_x(\theta ) \varvec{R}_y(\phi )\varvec{R}_z(\gamma ) r}) \end{aligned}$$

where elemental rotation matrices $\varvec{R}_x$, $\varvec{R}_y$, and $\varvec{R}_z$ are defined as

2 $$\begin{aligned} \varvec{R}_x= &   \begin{bmatrix} 1 &  0 &  0 \\ 0 &  \cos \theta &  -\sin \theta \\ 0 &  \sin \theta &  \cos \theta \end{bmatrix}, \varvec{R}_y = \begin{bmatrix} \cos \phi &  0 &  -\sin \phi \\ 0 &  1 &  0 \\ \sin \phi &  0 &  \cos \phi \end{bmatrix},\nonumber \\ \varvec{R}_z= &   \begin{bmatrix} \cos \gamma &  -\sin \gamma &  0 \\ \sin \gamma &  \cos \gamma &  0 \\ 0 &  h &  1 \end{bmatrix} \end{aligned}$$

The angle $\gamma$ represents the gantry angle and varies from $3.6^{\circ }$ to $360^{\circ }$ with an interval of $3.6^{\circ }$. The other rotation angles, denoted as $\theta$ and $\phi$, represent the couch angles and range from $0^{\circ }$ to $35^{\circ }$. They consist of a total of 36 angle combinations, given by:

$$\begin{aligned} {(\theta , \phi )}= {(0^{\circ },0^{\circ }), (1^{\circ },1^{\circ }),\ldots ,(35^{\circ },35^{\circ })} \end{aligned}$$

#### Step 3: projection

To simulate two-dimensional (2D) sequential fluoroscopic images, DRRs are computed through the forward projection of each rotated CT data into a 2D image plane. A ray tracing approach is used for DRRs generation in this study. The projection operation can be represented by:

3 $$\begin{aligned} \varvec{I}(i,j,p)=f(\varvec{V}_{\text {CT}, p, \theta , \phi , \gamma }(\varvec{r})), \end{aligned}$$

where $\varvec{I}(i,j,p)$ is the DRRs, *i* and *j* are coordinates of the image $\varvec{I}$, and $f(\cdot )$ is a function of projection operation. No motion artifacts or imaging noise are introduced during the projection process.

**Fig. 3:**
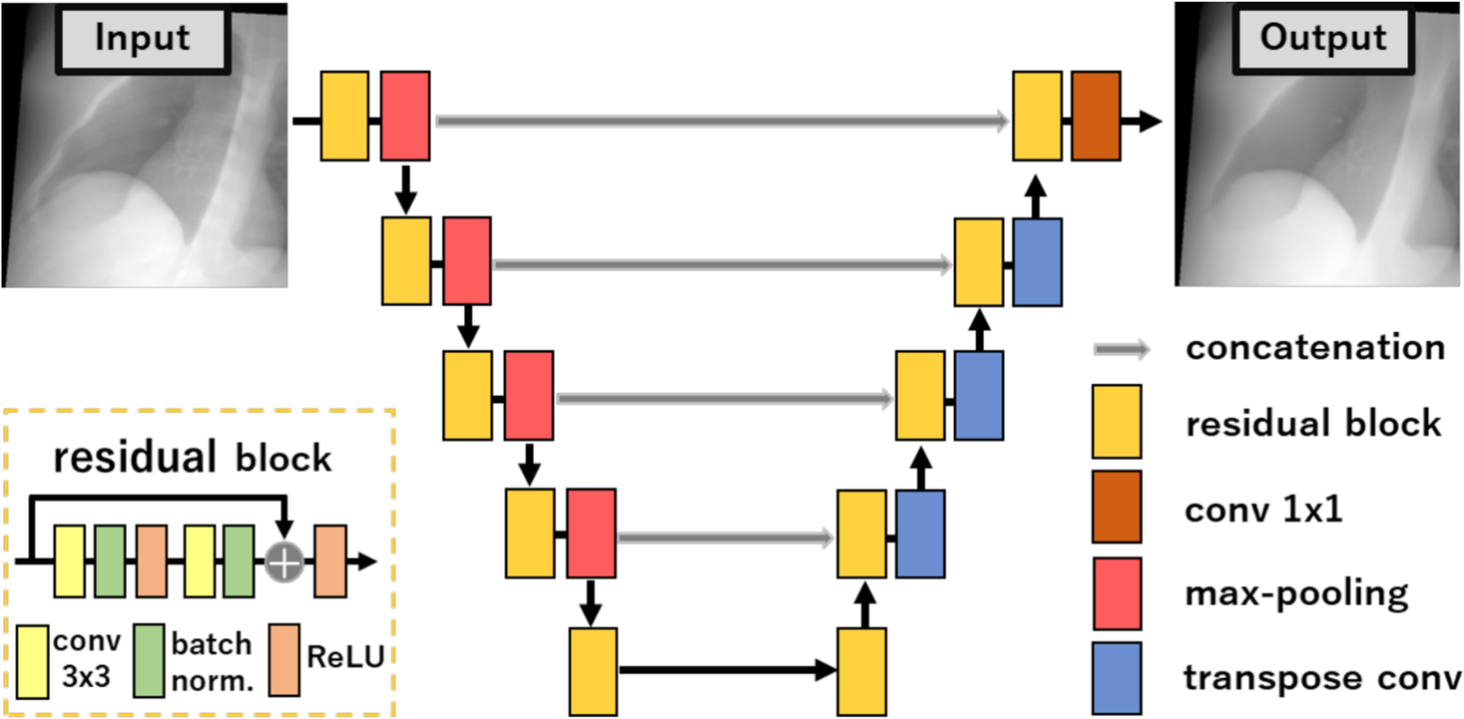
The schematic of residual U-net model

#### Step 4: subtraction

To obtain the DES image, the subtraction between the high-energy ($\varvec{I}_{\text {HE}}$) and low-energy ($\varvec{I}_{\text {LE}}$) images is computed using the following equation:

4 $$\begin{aligned} \ln \varvec{I}_{\text {DES}} = \ln \varvec{I}_{\text {HE}} - \omega \cdot \ln \varvec{I}_{\text {LE}} \end{aligned}$$

Here, $\varvec{I}_{\text {DES}}$ represents the DES image. By adjusting the weight coefficient $\omega$, components extracted from DES images can be generated. In this study, the high-energy image $\varvec{I}_{\text {HE}}$ is utilized as the SE image during model training, as it corresponds to the energy level used in actual x-ray imaging during treatment (e.g., 120 kVp). Since the soft-tissue enhanced DES images are more suitable for tumor tracking, the weight was set to $\omega =0.5$ based on the energy levels 120 and 60 kVp and prior research by Brody et al. [25].

#### Step 5: data augmentation

The final step involves performing geometric transformations on the SE images $\varvec{I}_{\text {SE}}$ and subtracted images $\varvec{I}_{\text {DES}}$ to simulate different imaging regions and account for variability in image scale and anatomy. In this study, we apply scaling, cropping, and padding techniques to both $\varvec{I}_{\text {SE}}$ and $\varvec{I}_{\text {DES}}$ in order to simulate different scales and regions of x-ray fluoroscopic images. Scaling is applied with random scale factors ranging from 0.8 to 1.2 to adjust the image size. Cropping is performed randomly to simulate partial field-of-view scenarios, typically cropping the image to 50–100% of its original size. Padding is applied as zero-padding to restore the image to its original dimensions when necessary. By augmenting the data in this way, we can ensure that our deep learning model is trained on a diverse set of images and can perform well on different types of input data. Using these techniques, we generate 36,000 sets of image $\varvec{I}_{\text {SE}, p, \theta , \phi , \gamma }(i,j)$ and $\varvec{I}_{\text {DES}, p, \theta , \phi , \gamma }(i,j)$.

### Deep learning model

A DL model is trained to learn the mapping from the SE image $\varvec{I}_{\text {SE}}$ as the input to its corresponding DES image $\varvec{I}_{\text {DES}}$ as the output. The DL model architecture used in this study is a residual U-net [26]. The original non-residual U-net architecture is a well-known convolutional neural network architecture for the image-to-image mapping task (e.g., segmentation) based on an encoder-decoder structure that allows it to capture the global context of the input image. This architecture can be crucial for effectively handling the DES prediction task, since the model needs to learn the difference of the overall structure or anatomy between SE and DES images. For instance, it allows the model to recognize variations in lung tissue density, lower airway patterns, and abnormalities in different regions of the lung. Moreover, the U-net’s multi-resolution approach can effectively handle the various image scales that SE images can have. The U-net model also incorporates skip connections that preserve fine details from the input image during the encoding process, which can be useful for faster convergence when the output contains resembled features with the input. In the residual U-net, by replacing conventional convolutional layers with residual convolutional layers, the training process can be eased because the skip connections contained in residual convolutional layers facilitate information propagation. The architecture of the model can be seen in Fig. 3 (see Appendix B for a brief ablation study on other architectural choices).

### DES synthesis

**Fig. 4:**
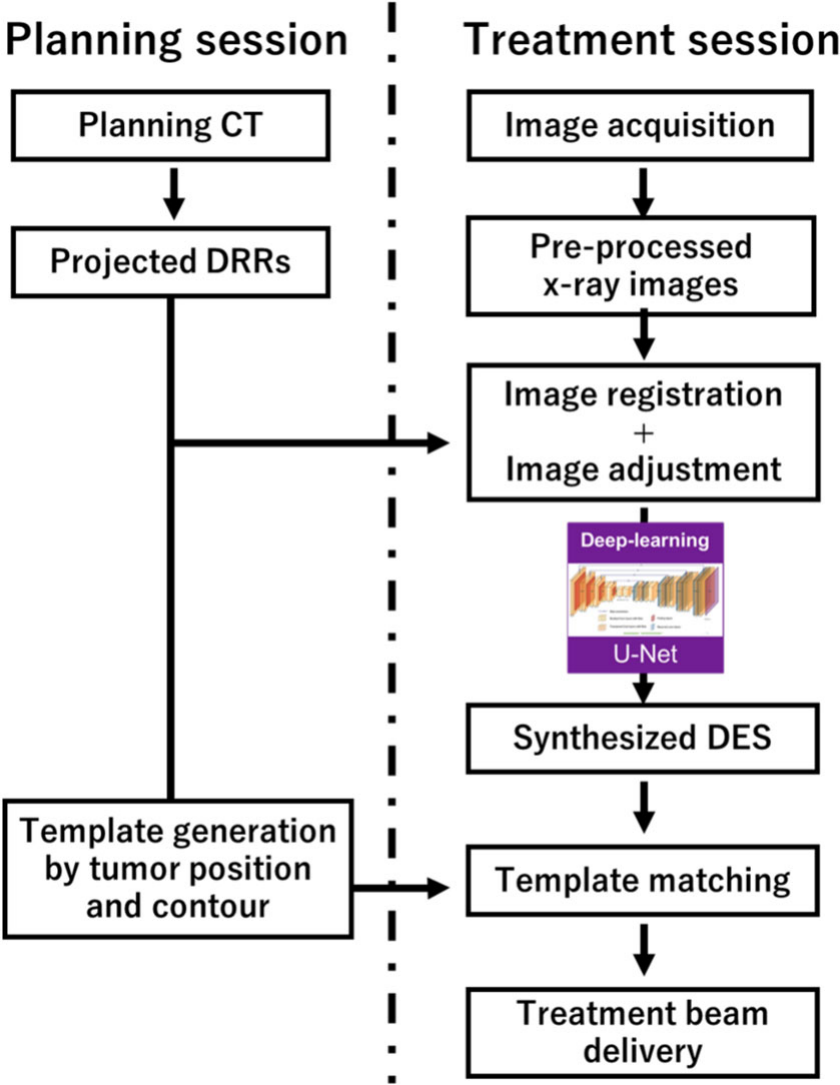
The workflow of DES synthesis in the treatment session

To demonstrate the application of our proposed method within clinical treatment sessions, we investigate its performance on clinical x-ray images. It is crucial to note that the model has been primarily trained on digitally reconstructed radiographs (DRRs), resulting in a greater proficiency in translating DRRs rather than clinical x-ray fluoroscopic images acquired during treatment. To bridge the gap between training and testing data, we have observed notable distinctions between geometrically registered DRRs and clinical x-ray fluoroscopic images, particularly in terms of contrast, spatial resolution, and soft tissue textures. In this study, a workflow is presented to adjust each x-ray fluoroscopic image to resemble a DRR-like image as depicted in Fig. 4.

In the planning session depicted in this workflow, DRR projections are first generated from planning CT data to match the imaging angles of each treatment fraction and verify the patient’s position for setup. During the treatment session, x-ray fluoroscopic images are obtained using an on-board imager. To verify and adjust the DRR position aligned with the target imaging area, affine registration is performed using ITK 4.2.0 [27], ensuring proper geometric alignment with the acquired x-ray fluoroscopic images (see Appendix C for the sensitivity analysis on the registration). This process incorporates translation, rotation, and scaling parameters to ensure precise alignment between DRRs and x-ray fluoroscopic images. After registration, histogram matching [28] is employed to align the image intensity with the reference DRR. Additionally, a $5\times 5$ median smoothing filter is applied to refine the spatial resolution and soft tissue textures of the clinical images, making them more close to the reference DRRs. These pre-processing steps ensure that the clinical images are standardized for subsequent synthesis. Finally, the adjusted clinical images are used as input to the trained model to predict DES images. The output DES images can then be directly utilized for tumor tracking.

### Tumor tracking

A template matching technique based on normalized cross-correlation (NCC) to assess and compare the performance of tumor tracking for SE and synthesized DES images. The NCC-based template matching is commonly employed in MTT techniques [6, 7]. For a given search position of (*u*, *v*), the NCC value is computed using the following formula:

5 $$\begin{aligned} \text {NCC}(u,v)=\frac{\displaystyle \sum _{i=0}^{w-1} \sum _{j=0}^{h-1}(I(u+i,v+j)T(i,j))}{\displaystyle \sqrt{ \sum _{i=0}^{w-1} \sum _{j=0}^{h-1}(I(u+i,v+j))^2}\displaystyle \sqrt{\sum _{i=0}^{w-1} \sum _{j=0}^{h-1}(T(i,j))^2}} \end{aligned}$$

Here, *I*(*i*, *j*) and *T*(*i*, *j*) represent the target and template images, respectively, and *w* and *h* are the width and height of the template image.

## Experimental settings and dataset

We conducted one experiment to evaluate the effectiveness of DL model training using unseen artificial data. In another experiment of investigating the potential of DL-based DES synthesis in a clinical scenario, we evaluated tumor tracking performance using clinical x-ray data, and compared the SE images and the synthesized DES from the U-net model in the Part 4, as illustrated in Fig. 1.

### Deep learning model training

We implemented and trained the residual U-Net using the Keras 2.1.0 [29] deep learning framework. During training, the mean squared error (MSE) between the output and ground truth is used as the loss function:

6 $$\begin{aligned} \text {MSE} = \frac{1}{mn} \sum _{i=1}^m \sum _{j=1}^n (\varvec{I}_\text {DES}(i,j)-\hat{\varvec{I}}_\text {DES}(i,j))^2, \end{aligned}$$

where *m* and *n* are the numbers of rows and columns in both the output image $\hat{\varvec{I}}_{\text {DES}}(i,j)$ and the ground truth image $\varvec{I}_{\text{ DES }}(i,j)$. Adaptive moment estimation (Adam) is used as an optimizer, since it has a rapid convergence rate and high stability. The initial learning rate was set to 0.02 with linear learning rate decay, decreasing the learning rate to $1 \times 10^{-4}$ over the course of training. The model was trained for up to 200 epochs using one NVIDIA Quadro GV100 graphics processing unit (Nvidia, Santa Clara, CA, USA).

To evaluate the effectiveness of the trained model, we generated four unseen artificial cases with varying gantry angles ($\theta$, $\phi$), and the couch angle is ranged from $3.6^{\circ }$ to $360^{\circ }$. Each case consisted of 150 sequential images captured over five respiratory cycles with a spherical tumor of 10 mm diameter.

To quantitatively assess the quality of the synthesized DES images, we calculated the peak signal-to-noise ratio (PSNR) and structural similarity index (SSIM) values between the synthesized DES images and the reference DES images. The objective of this evaluation was to assess whether the model was effectively trained and capable of making accurate DES predictions in new cases.

**Table 1:** Summary of datasets used for tumor tracking experiments

#	Tumor location	Tumor diameter	Motion range	Gantry angle	Couch angle
1	Right lower	2.80 cm	5.06 mm	$180^{\circ }$	$0^{\circ }$
2	Right lower	1.38 cm	12.39 mm	$190^{\circ }$	$280^{\circ }$
3	Right upper	1.43 cm	3.03 mm	$190^{\circ }$	$280^{\circ }$
4	Right upper	1.46 cm	10.83 mm	$180^{\circ }$	$270^{\circ }$
5	Left upper	6.60 cm	19.73 mm	$0^{\circ }$	$0^{\circ }$
6	Left middle	1.09 cm	5.21 mm	$180^{\circ }$	$0^{\circ }$
7	Right upper	2.28 cm	4.17 mm	$180^{\circ }$	$0^{\circ }$
8	Left middle	1.17 cm	5.09 mm	$280^{\circ }$	$10^{\circ }$
9	Left upper	1.40 cm	4.95 mm	$200^{\circ }$	$290^{\circ }$
10	Right upper	1.51 cm	10.70 mm	$270^{\circ }$	$0^{\circ }$

### DES synthesis using clinical data

In order to assess whether synthesized DES can provide advantages for MTT, ten cases of clinical x-ray fluoroscopic image sequences were used to evaluate tumor tracking performance. The clinical data were acquired from patients who had received stereotactic body radiation therapy for lung cancer at Hirosaki University Hospital.^1^ Image acquisition was performed using an on-board imager on Varian Clinac iX (Varian Medical Systems, Palo Alto, CA, USA). Table 1 summarizes the details of the dataset.

### Evaluation methods of tumor tracking

One evaluation metric in our study is tracking error, which is measured by the root mean square error (RMSE) between the positions of the ground truth and the template matching. The RMSE is computed as follows:

7 $$\begin{aligned} \text {RMSE} = \sqrt{\frac{1}{n}\sum _{t=1}^{n}((x_g(t) - x(t)) ^2 + (y_g(t) - y(t))^2)} \end{aligned}$$

where *x*(*t*) and *y*(*t*) represent the positions where the template is matched, and $x_g(t)$ and $y_g(t)$ represent the positions of the ground truth. To obtain the ground truth, we manually contoured the tumor in each frame and calculated its centroid.

The tracking success rate (TSR) is another evaluation metric that measures the percentage of frames with successful tracking out of the total number of frames in the testing case. A success of tracking is defined as when the Euclidean distance of the tracking error is below 25% of the maximum tumor movement range in the current case.

**Fig. 5:**
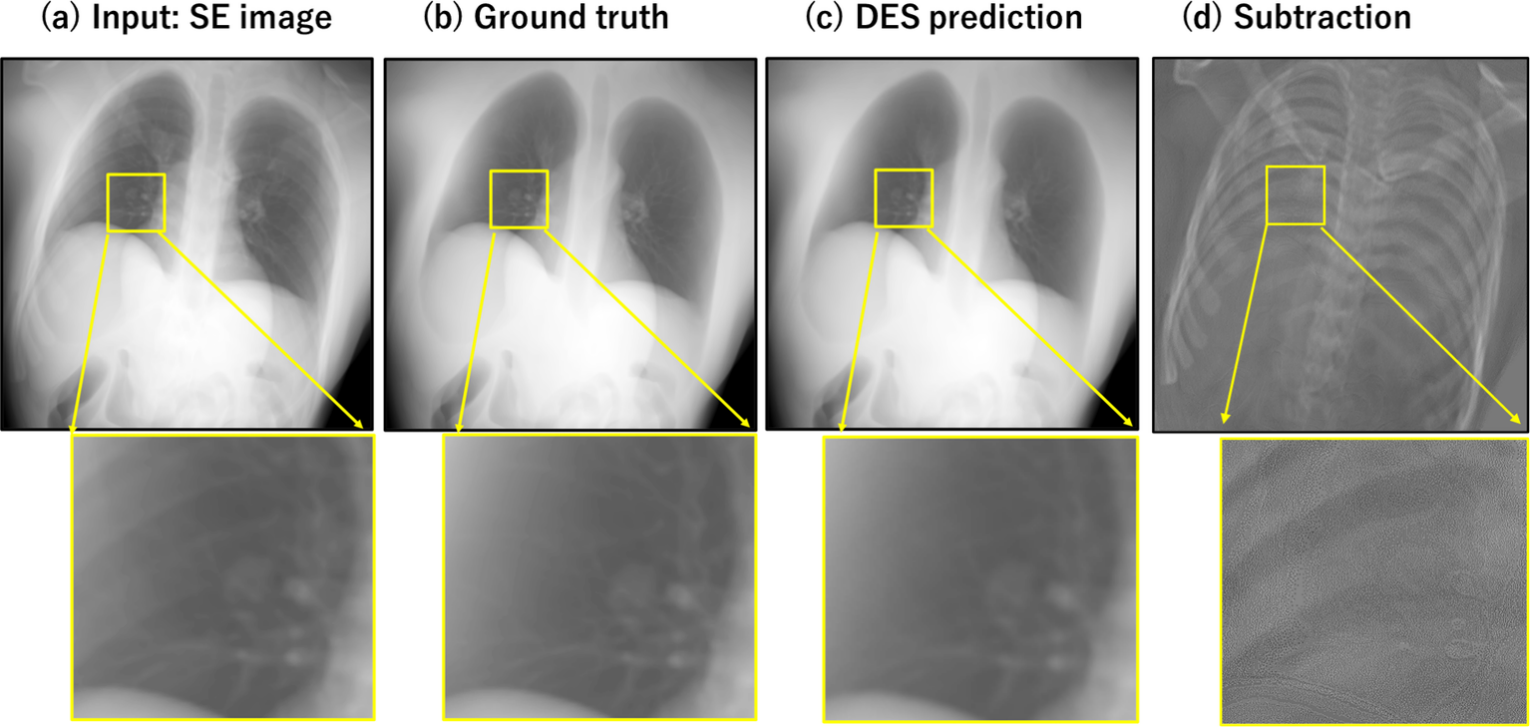
Examples of input and output for the trained model. Input image (a) is unseen SE image. (b) is the ground truth of the corresponding soft-tissue enhanced DES image. (c) is the output of the DES image from (a). (d) is the difference between (a) and (c), which corresponds to the suppressed components by U-net model. The yellow rectangle shows a lung nodule overlapped by the rib bone in each image

## Results

### Results of DL model training

Figure 5 shows a comparison between the input SE image (a), the ground truth of DES (b), the output DES image (c) obtained from testing artificial data using the U-net model, and the difference (d) between the input (a) and the output (c). We can observe that the U-net model effectively removed the entire bone structure in the output (c), while preserving the soft tissue. Further, the output (c) looks very similar visually to the ground truth (b). The difference image (d) reveals the components that have been removed during the DES synthesis process, displaying a complete bone structure without any soft tissue. Notably, the DES prediction removed the overlapped rib bones in the input image, thereby making the lung nodule (in the yellow rectangle) more visible than in the original SE images.

Table 2 presents a summary of the quantitative evaluation in terms of PSNR and SSIM. The PSNR values, exceeding 40 dB, reflect minimal distortion when compared to the ground truth, with the highest PSNR reaching 48 dB for 8-bit images. Furthermore, all SSIM values surpass 0.99, signifying minimal structural distortion in the predictions. Based on these results, it can be concluded that the model was successfully trained for DES prediction in the artificial datasets.

### Results of DES synthesis

**Fig. 6:**
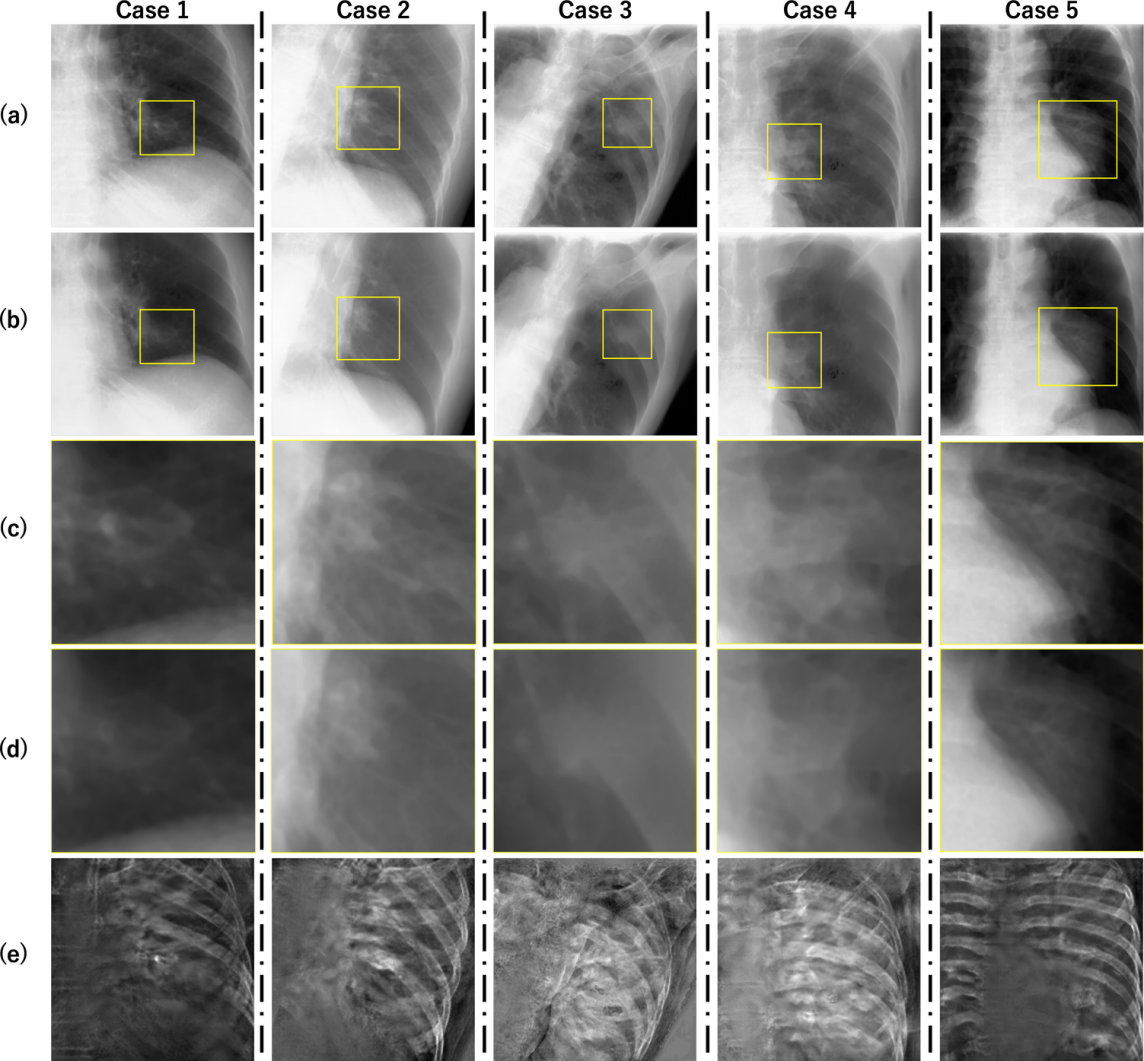
Comparison of DES synthesis results for cases 1 to 5. Each column represents a single case. The top row (a) shows the input SE image with a yellow bounding box indicating the tumor location. The second row (b) displays the corresponding output DES image with the same tumor region highlighted. The third row (c) shows a zoom-in of the tumor region in (a), while the fourth row (d) shows a zoom-in of the corresponding region in (b), to better illustrate the tumor enhancement. The bottom row (e) presents the subtraction between (a) and (b), revealing the suppressed components

**Fig. 7:**
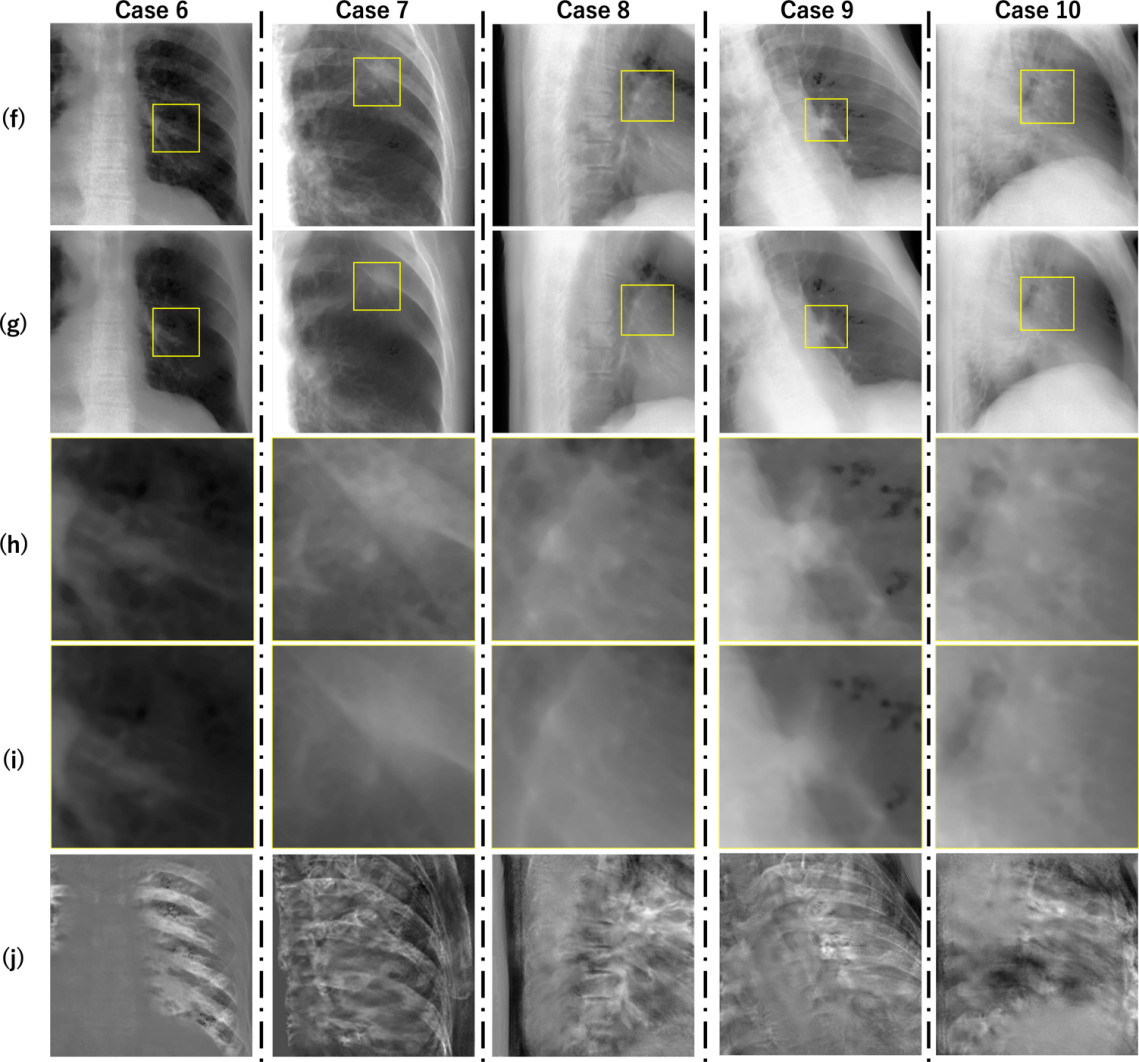
Comparison of DES synthesis results for cases 6 to 10. Each column represents a single case. The top row (f) shows the input SE image with a yellow bounding box indicating the tumor location. The second row (g) displays the corresponding output DES image with the tumor region marked similarly. The third row (h) presents a zoom-in of the tumor region in (f), and the fourth row (i) shows a similar zoom-in of the same region in (g) to better illustrate the tumor expansion. The bottom row (j) presents the subtraction between (f) and (g), revealing the suppressed components

Figures 6 and 7 present a visual comparison between input SE images and output DES images of ten clinical cases. The randomly selected SE images are shown in the upper row (a) and (f) with a yellow bounding box indicating the tumor region, while its corresponding output of DES prediction are shown in the second row (b) and (g). The zoomed-in views of the tumor region in SE and DES are presented in the rows (c), (d) and (h), (i), respectively, to highlight changes in tumor visibility. The subtractions between input and output are displayed in the bottom row (e) and (j), which represent the components suppressed by the DES prediction. The results indicate that: (1) the rib bone components are partially suppressed in almost all cases; however, the extent of suppression was inadequate, as some bone structures such as the spine remained visible, as seen in the subtraction images; (2) some DES predictions improved the visibility of the target tumor; (3) in some cases, details of soft tissue were lost. For example, in case 5 (Fig. 6), the rib bone structures were nearly entirely eliminated, leading to an enhancement in tracking performance. However, in cases 3, 4, 7, 9, and 10, although rib bone structures were partially suppressed, the textures and edges of the target tumor were lost.

**Table 2:** The quantified PSNR and SSIM values of the synthesized DES images in four artificial datasets

Couch angles ($\theta$,$\phi$)	PSNR [dB]	SSIM
$( 9^{\circ }, 28^{\circ })$	40.168	0.993
$(17^{\circ }, 5^{\circ })$	42.106	0.993
$(20^{\circ }, 32^{\circ })$	42.136	0.993
$(25^{\circ }, 21^{\circ })$	44.089	0.993

### Results of tumor tracking

Table 3 summarizes the RMSE tracking errors and tracking success rate (TSR) on SE images and synthesized DES images from the ten cases with 95% confidence intervals (CI). This table shows that more minor tracking errors were achieved on the synthesized DES than on the raw SE in all cases. The bootstrap hypothesis testing (*p*=0.05) rejected the null hypothesis $H_0: \text {RMSE}_{\text {SE}} = \text {RMSE}_{\text {DES}}$ in 9/10 and $H_0: \text {TSR}_{\text {SE}} = \text {RSR}_{\text {DES}}$ in 8/10 cases respectively (marked with asterisks in Table 3), indicating statistically significant difference in tumor tracking performance for the synthesized DES images compared with SE images. The tracking error (RMSE) of the case on the SE images was 1.80 mm (CI: 1.17–2.38 mm). On the synthesized DES images, the error decreased to 1.65 mm (CI: 1.11–2.10 mm). The case 5 showed the most significant improvement, with a decrease from 3.05 to 2.23 mm, representing a 27% reduction in errors.

**Fig. 8:**
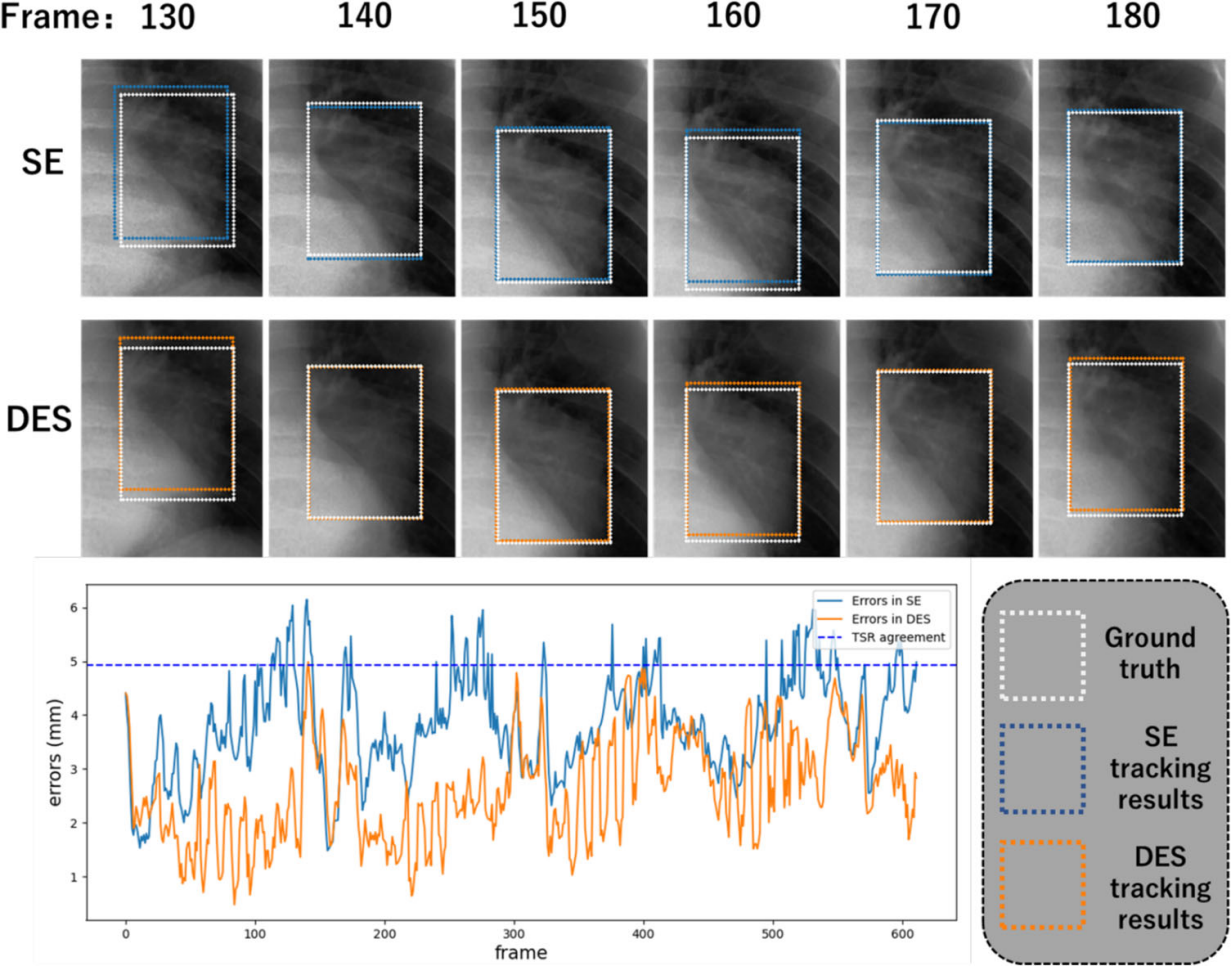
Comparison of tracking results and TSR in SE vs. DES images for case 5. The upper panel compares the tracking results in SE images (blue rectangle) and synthesized DES images (orange rectangle) with the manually contoured ground truth (white rectangle) for case 5 during a respiratory cycle from frame 130 to 180. The lower panel shows the tracking errors in SE and DES images with an agreement line for successful tracking

Regarding TSR, there is also 90% case improved with the dataset-average improvement from 50.2% (CI: 27.54–71.44%) to 54.9% (CI: 31.24–77.36%) when using synthesized DES as target images. Among the cases, the most significant improvement is case 5, which improved from 85.8 to 98.9%. Figure 8 shows the tracking results in case 5, with the DES tracking errors (orange line) in this case are mostly below the agreement line, indicating improved tracking performance compared to SE images. The only case that did not show improvement is case 10, which decreased from 86.3 to 84.6% due to the loss of soft tissue texture in the synthesized DES images. A summary of the experimental results, for both artificial and clinical datasets, is presented in Table 4 (Appendix A).

## Discussion

**Fig. 9:**
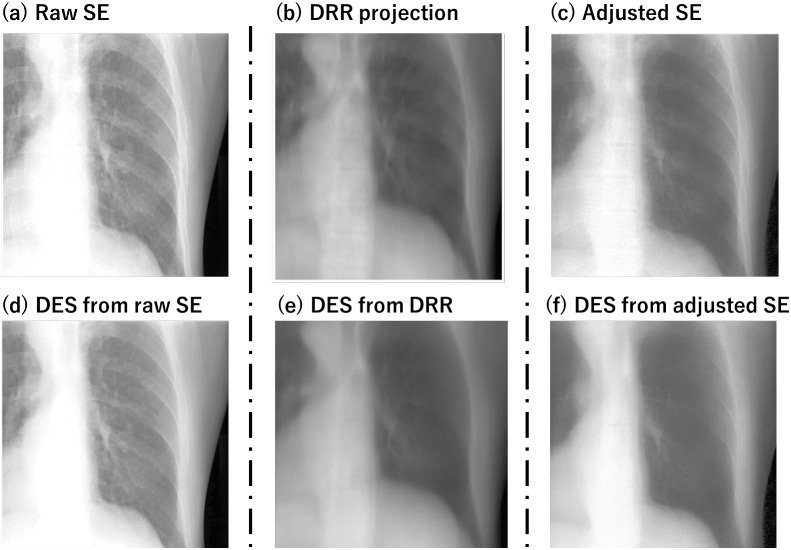
Comparison of DES prediction from raw SE image, DRR projection and adjusted SE image. (a) is the SE image from dataset#3, (b) is generated DRRs, and (c) is adjusted SE image from the same data. (d), (e), and (f) are DES predictions of (a), (b), and (c), respectively

In this study, we used U-net model to predict DES images from kV x-ray images. To deal with the insufficient DES dataset, we utilized artificial data from a digital phantom to train the U-net model. The results showed that the U-net model can eliminate some rib bones in the synthesized DES images, which is desirable for improving tumor tracking. However, the spinal bones were not eliminated in the DES images. Additionally, soft tissue was partly removed, which is not ideal as it causes the loss of details of image texture, including the edge of the target tumor. Consequently, the insufficient performance of DES synthesis leads to a slight but insignificant improvement in tracking accuracy and success rate. The main possible reason is the differences between the artificial and clinical data, such as the anatomical variations, image contrast, and noise. To narrow the differences, we adopted image adjustment to each clinical data as mentioned in Subsection 2.3. Here, we give an example to show the differences and the consequential outcome in Fig. 9. The upper row is the input, and the bottom row is the corresponding DES prediction of the input. As evident from the prediction (d), there are minimal changes compared to the input SE x-ray image (a). In contrast, in the prediction (e) derived from the DRRs projected from the same patient’s CT data, the bone structure, including the spine, is noticeably suppressed. Since our model is trained on DRRs, it may struggle with x-ray images featuring higher luminance and spatial resolution. After undergoing image adjustment, the adjusted x-ray images (c) closely resemble DRRs (b), and the resulting output (f) exhibits improved predictions compared to (d).

**Table 3:** Tumor tracking error (RMSE) and tracking success rate (TSR) on SE images and DES images (case 1–10) with 95% confidence intervals (CI)

#	SE (Latency: 5 ms)		DES (Latency: 55 ms)	
	RMSE (mm)	TSR (%)	RMSE (mm)	TSR (%)
1	3.22	33.6	$\varvec{3.02}$ ${*}$	$\varvec{38.4 }$ ${*}$
2	1.24	32.9	$\varvec{1.20}$ ${*}$	$\varvec{42.0 }$ ${*}$
3	0.61	52.2	$\varvec{0.60}$	$\varvec{57.5 }$ ${*}$
4	1.85	89.6	$\varvec{1.78}$ ${*}$	$\varvec{91.4 }$ ${*}$
5	3.05	85.8	$\varvec{2.23}$ ${*}$	$\varvec{98.9 }$ ${*}$
6	1.83	5.9	$\varvec{1.73}$ ${*}$	$\varvec{8.1 }$ ${ *}$
7	1.20	14.6	$\varvec{1.19}$ ${*}$	$\varvec{16.8 }$ ${*}$
8	0.82	74.1	$\varvec{0.76}$ ${*}$	$\varvec{85.4 }$ ${*}$
9	1.49	19.9	$\varvec{1.45}$ ${*}$	$\varvec{19.9 }$
10	2.45	$\varvec{86.3}$	$\varvec{2.10}$ ${*}$	$\varvec{84.6 }$
Average	1.80	50.2	$\varvec{1.68}$	$\varvec{54.9}$
95% CI	1.31–2.36	30.91–68.86	1.22–2.09	34.95–74.79

$*$ indicates a statistically significant difference between SE and DES For SE images, only preprocessing (smoothing filter and histogram matching: 5 ms) is required. For DES images, total latency includes preprocessing (5 ms) and U-Net inference (50 ms), resulting in an end-to-end latency of approximately 55 ms

Despite the improvement brought by image adjustment, the adjustment is still insufficient for DES prediction, leading to uncompleted bone suppression. Meanwhile, the lost information and spatial resolution during smoothing are not suitable for the subsequent application process, such as tumor tracking. One major factor contributing to these limitations is the simplified nature of our training dataset, which consists of artifact-free synthetic images with limited anatomical variability. In contrast, clinical fluoroscopic images frequently exhibit complex artifacts such as beam hardening, scatter, and patient motion, which are not adequately represented in our current training dataset (Table 4).

To bridge this gap, future work will focus on enhancing the realism and diversity of the training data by incorporating simulated motion artifacts, imaging noise, and a broader range of anatomical structures. Regarding anatomical variability, our current DES data generation process relies on CT volumes simulated from digital phantoms, To expand anatomical diversity, future work may explore methods proposed by Segars et al. [30], such as using diffeomorphic registration to align template XCAT phantoms with patient-specific anatomies, thereby introducing more realistic variations in organ shapes and body structures. Compared to adding variations through digital phantom simulation, the incorporation of clinical dual-energy CT (DE-CT) data could further enhance anatomical diversity by providing paired DE-CT data with more realistic and heterogeneous tissue compositions. Such data can offer valuable material-specific information and more accurate modeling of attenuation properties, which may improve both anatomical realism and soft-tissue contrast in the synthesized images. Regarding achieving realistic artifacts and imaging noise, recent developments in simulation frameworks could support these goals. For instance, DeepDRR [31] suggest a promising direction for simulating clinical artifacts more realistically through material decomposition and scatter estimation, which could help simulate beam hardening and other complex artifacts. Another study, fastCAT [32] proposed a physics-based simulation framework utilizing pre-computed Monte Carlo scatter kernels and detector-specific response modeling to efficiently generate high-fidelity CBCT projections under clinically realistic conditions. In addition to these purely synthetic methods, integrating data from physical phantom experiments conducted under clinical DE imaging scenarios, which could provide another valuable source of anatomical and artifact variability. To bridge the gap between synthetic and clinical data domains, unsupervised domain adaptation methods may be explored. For example, adversarial training, style transfer, or contrastive representation learning could be employed to transform DRR-like synthetic images into more clinically realistic x-ray appearances, or vice versa. These techniques aim to reduce the domain discrepancy between training and deployment conditions, thereby improving model generalization and performance on real clinical data. We believe that these enhancements will enable the model to better preserve soft-tissue information while maintaining effective bone suppression, ultimately improving the robustness and clinical utility of the synthesized DES images.

Since there are no DES images clinically obtained by kV x-ray imaging during radiation therapy, the image quality of the synthesized DES images cannot be quantitatively validated. Instead of the quality validation on the clinical dataset, the performance of tumor tracking was directly compared to evaluate the effect of the DES synthesis in this study. For evaluating the tumor motion tracking results, the ground truth was determined through manual contouring under the guidance of a medical physicist, which can be subjective and uncertain. The contouring variability in how to interpret and segment images can lead to inconsistencies and errors in the ground truth, potentially affecting the evaluation and interpretation of the results. To address this limitation, future work should involve multiple annotators labeling a subset of cases to comprehensively assess inter-observer variability. The evaluation needs to quantify tumor delineation differences using volume overlap and Dice coefficient metrics. Additionally, it is also important to analyze tumor motion trajectory discrepancies through center-of-mass comparisons and trajectory deviation statistics. Finally, the impact of annotation variability on tracking performance will be evaluated by measuring RMSE differences between SE and DES conditions in the template-matching method. This systematic analysis will provide crucial insights into tracking robustness and inform annotation protocol refinements for improved clinical applicability.

Moreover, the sample size of clinical datasets used in this study was relatively small (10 cases), which is a limitation on the generalizability of the findings despite efforts to include diverse tumor sizes and motion patterns. Validating the proposed approach using a larger and more anatomically diverse patient cohort is future work. In addition to expanding the clinical dataset, future work needs to incorporate phantom-based experimental data acquired under realistic imaging conditions, which would also allow direct comparison with classical tumor enhancement methods under controlled settings. Recent research has shown the potential of phantom-based dual-energy imaging to enhance soft-tissue visibility and simulate clinical artifacts [14, 16, 17]. By integrating such data into our training and evaluation pipeline, we aim to improve not only the realistic anatomical representation of the synthesized DES images but also their performance in downstream tasks such as markerless tumor tracking.

Real-time capability is an important factor for the image processing techniques in tumor tracking to achieve accurate radiation delivery to the moving tumor. Currently, the standard of kV x-ray imaging captures 15 frames per second. That is, the time interval between two frames is about 66.7 ms. The proposed method used in this study completed the DES prediction within 50 ms per frame using one NVIDIA Quadro GV100 graphics processing unit (Nvidia, Santa Clara, CA, USA). When combined with the 5 ms preprocessing time (for histogram matching), the total processing time of 55 ms per frame demonstrating real-time capability. In addition, the preprocessing process, including affine registration and histogram matching, is fully automated once the reference DRR and initial fluoroscopic frame are defined. Affine registration is performed only once between the reference DRR and the first fluoroscopic frame, requiring approximately 1 s to compute the initial transform, while histogram matching adds approximately 5 ms per frame. Furthermore, the primary role of registration in our workflow for the DES synthesis during treatment is to facilitate histogram matching rather than achieve perfect anatomical alignment, making the pre-processing relatively insensitive to minor misregistration.

In this feasibility study, we employed residual shortcuts and a standard U-Net backbone to focus on demonstrating the feasibility of DES synthesis from x-ray fluoroscopy. Nevertheless, future work will explore a broader range of architectural enhancements (e.g., deeper layers, attention mechanisms), alternative activation functions, and hyperparameter tuning. A comprehensive ablation study needs to be performed to assess the individual impact of each component and learning rate strategy on overall performance. In addition, incorporating more diverse data augmentation strategies, such as noise injection and brightness shifts, will be important to improve model generalizability. Specifically, brightness shifts can enhance the robustness of downstream pre-processing steps like histogram matching, particularly when combined with more realistic simulations of clinical artifacts.

In summary, our results suggested that a DL-based DES synthesis model for x-ray fluoroscopy can be built through a simulated phantom dataset-based training strategy and has the potential to enhance target visibility as well as improve tumor tracking accuracy in real-time. Future work should focus on addressing the limitations of our study, including the generation of more realistic high-energy and low-energy DRRs, as well as the development of an effective and quantitative evaluation method to assess DES prediction performance.

## Conclusion

We presented a DL-based method to synthesize DES images from SE fluoroscopy using a residual U-net network and demonstrated the possibility and effectiveness of DL-based DES synthesis for markerless tumor tracking. When testing on the artificial data, most overlapped rib bones in the input image were suppressed in the synthesized DES images, achieving an SSIM of 0.993 and a PSNR of more than 40 in four pairs of couch angles. When testing on the clinical data, the synthesized DES suppressed bone structures and improved the tumor tracking performance. The average tracking error was reduced from 1.80 to 1.68 mm for RMSE, and the tracking success rate increased from 50.2 to 54.9%. These results suggest that DL-based DES synthesis has potential in utilizing DE imaging without specialized hardware for radiation therapy in lung cancer treatment.

## Data Availability

The artificial data are not publicly available since they were generated using licensed software, and the clinical data cannot be publicly shared from the authors for privacy reasons.

## Appendix A: Summary of quantitative results

**Table 4:** Unified quantitative results on artificial and clinical datasets

Dataset	Case	PSNR [dB]	SSIM	RMSE (mm)	TSR (%)	Latency [ms]
	$(9^{\circ }, 28^{\circ })$	40.168	0.993	–	–	–
Artificial	$(17^{\circ }, 5^{\circ })$	42.106	0.993	–	–	–
	$(20^{\circ }, 32^{\circ })$	42.136	0.993	–	–	–
	$(25^{\circ }, 21^{\circ })$	44.089	0.993	–	–	–
	$(25^{\circ }, 21^{\circ })$	44.089	0.993	–	–	–
	1	–	–	3.02$^*$/3.22	38.4$^*$/33.6	
	2	–	–	1.20$^*$/1.24	42.0$^*$/32.9	
	3	–	–	0.60/0.61	57.5$^*$/52.2	
	4	–	–	1.78$^*$/1.85	91.4$^*$/89.6	
	5	–	–	2.23$^*$/3.05	98.9$^*$/85.8	
Clinical	6	–	–	1.73$^*$/1.83	8.1$^*$/5.9	55/5
	7	–	–	1.19$^*$/1.20	16.8$^*$/14.6	
	8	–	–	0.76$^*$/0.82	85.4$^*$/74.1	
	9	–	–	1.45/1.49	19.9/19.9	
	10	–	–	2.10$^*$/2.45	84.6/86.3	
Average		–	–	1.68/1.80	54.9/50.2	
95% CI		–	–	1.22–2.09/1.31–2.36	34.9–74.7/30.9–68.9	

$^*$ Statistically significant improvement compared to SE image. Format: DES / SE In artificial datasets, synthesized DES images are compared to ground truth using PSNR/SSIM to assess image quality. In clinical datasets where no DES ground truth is available, evaluation is based on tumor tracking accuracy derived from DES/SE comparisons

## Appendix B: Ablation study on activation functions and network layer depth

We conducted a simple ablation study to evaluate two key architectural choices: activation functions and network depth. Activation functions: the originally used ReLU and the smoother GELU activation. Network depth: U-Net architectures with 4 to 6 encoder–decoder levels, where the baseline model employs 5 levels. All experiments were conducted under the same training conditions as the original study and tested on the unseen artificial dataset (couch and collimator angles of 6°and 20°). The evaluation metrics include PSNR and SSIM.

**Table 5:** Performance comparison across activation functions and network depths

Activation	Depth	PSNR (dB)	SSIM
ReLU	4	39.4909	0.9827
ReLU	5	41.2411	0.9875
ReLU	6	41.5744	0.9884
GELU	4	38.8303	0.9795
GELU	5	39.5950	0.9823
GELU	6	39.6496	0.9819

According to Table 5, the 6-level architecture with ReLU achieved the best (PSNR=41.5744 dB and SSIM=0.9884) among the other architectural conditions. However, its performance was only marginally different from the second best of the original 5-level architecture with ReLU (PSNR=41.2411 dB and SSIM=0.9875), while the 4-level architecture with ReLU had the reduced performance, especially in PSNR. For the architecture with GELU, 5- and 6-level architectures only slightly outperformed the 4-level architecture with ReLU, and the difference between 5- and 6-level architectures with GeLU was very small in PSNR (39.6496 and 39.5950) and even had a marginally lower SSIM (0.9819 and 0.9823). In terms of activation functions, ReLU consistently outperformed GELU across all tested depths, with more significant performance gaps at greater depths (about 2 dB higher PSNR at depth 6). These results suggest that the choice of ReLU represents a better configuration. Regarding the depth level, the performance increases with the level in both ReLU and GeLU, as the 4-level ones showed lower performances than higher depth levels. However, the only small difference between 5-level and 6-level was found, suggesting that the original 5-level architecture is a reasonable choice for balancing performance and model complexity (training time and cost). Although the original U-net architecture (i.e., 5-level depth with ReLU) could be a reasonable configuration choice,

## Appendix C: Sensitivity analysis on misregistration in the preprocessing

The primary role of registration in preprocessing is to automatically align the DRR with the x-ray fluoroscopic images for histogram matching. To investigate the impact of the misregistration,, we conducted an additional experiment with three scenarios: (1) Original affine registration as implemented in our pipeline; (2) Affine registration after introducing perturbations, including (a) adding Poisson noise with an intensity scaling factor of 0.3 and (b) applying a random 10°rotation; (3) Non registration applied. Since ground-truth deformation fields are unavailable, we evaluated the effect of these registration scenarios by computing similarity metrics (Normalized cross-correlation (NCC)) between the original affine-registered images and those obtained in the other two scenarios. Additionally, we examined the influence of these registration scenarios on the subsequent tumor tracking experiments by calculating its tracking errors (RMSE), using the same metric with the original experiment in the manuscript. All experiments were performed using a representative clinical case (Case 4).

**Table 6:** Impact of registration quality on pipeline performance

Registration scenario	NCC	RMSE (mm)
Original affine	1.000	1.78
Affine with perturbations	0.832	1.81
No registration	0.624	1.93

As shown in Table 6, the NCC between affine-registration and affine-registration with noise and rotation is 0.832, suggesting the effect of misregistration, but the tracking error (RMSE) shows a small change (1.78 to 1.81). Even without registration, which results in greater misalignment (NCC 0.624), the RMSE remains largely unchanged. These findings suggest the pipeline’s robustness to perturbations and misregistration.

## Footnotes

1 This study was approved by the committee of Medical Ethics of Hirosaki University School of Medicine, Hirosaki, Japan.

## Code availability

Code is available from the corresponding author upon reasonable request.
